# Editorial: Circadian rhythm, athletic performance, and physical activity

**DOI:** 10.3389/fphys.2024.1466152

**Published:** 2024-07-31

**Authors:** Lucia Castelli, Jamie Hugo Macdonald, Pasquale Fabio Innominato, Letizia Galasso

**Affiliations:** ^1^ Department of Biomedical Sciences for Health, University of Milan, Milan, Italy; ^2^ Institute for Applied Human Physiology, Bangor University, Bangor, United Kingdom; ^3^ Oncology Department, Ysbyty Gwynedd, Betsi Cadwaladr University Health Board, Bangor, United Kingdom; ^4^ Cancer Research Centre, Warwick Medical School, University of Warwick, Coventry, United Kingdom; ^5^ UPR “Chronotherapy, Cancers and Transplantation”, Faculty of Medicine, Paris-Saclay University, Villejuif, France

**Keywords:** circadian rhythm, athletic performance, physical activity, exercise, chronotype, physiological responses

## Introduction

Human nature has time-related elements, with rhythms present at various levels of organization. Among these, circadian rhythms are the most extensively studied. The term “circadian” comes from the Latin words “circa,” meaning “about,” and “diem,” meaning “day” or of about 24 h. It refers to human organisms’ functions that have cycles roughly around 24 h (20–28 h). These rhythms are driven by a core set of circadian clock genes that interact in a feedback loop, determining the periods and oscillations of these cycles (Roenneberg et al., 2003; Bonaconsa et al., 2014; Janoski et al., 2024).

Sleep is an essential human process, and the alternation of sleep and wake states, the sleep-wake cycle, is a physiological rhythm temporally controlled by the body clock (Franken and Dijk, 2024). In addition to rhythmic hormone release, the body clock, and thus the sleep-wake cycle, is primarily regulated by the alternation of day and night, as well as other factors like meal timings and social routines (Zerón-Rugerio et al., 2021). Thus, the orchestration of circadian rhythms, including the sleep-wake cycle, is coordinated by both internal/endogenous factors and external/exogenous cues.

Furthermore, circadian rhythms in humans manifest as a complex phenotype influenced by various genetic factors that define an individual’s chronotype. Within individuals, there are differences in the timing of bodily functions, and between individuals, there are variations in diurnal preference, such as those who are active early (morningness) and those who are active later (eveningness). This trait should be evaluated not only in its own right but also in how it affects responses to external schedules. Depending on their inherent circadian rhythm features, people have different preferred times for sleep and activity, which is captured by the concept of chronotype (Adan et al., 2012). Additionally, these inter-individual differences impact on the timing of exposure to external synchronisers.

Three distinct chronotypes have been defined: Morning-types (M-types) and Evening-types (E-types), each further divided into extreme and moderate subtypes, and Neither-types (N-types). A person’s chronotype exists on a spectrum between morning and evening preferences. Those without a strong circadian preference are classified as N-types, as they display intermediate characteristics (Horne and Ostberg, 1976; Adan et al., 2012).

The influence of circadian rhythms has been explored in the context of physical activity and athletic performance, based on the assumption of a bidirectional connection between them. Many biological and behavioral functions that affect physical exercise follow regular circadian patterns. Numerous physiological variables impact physical performance, and understanding their daily fluctuations requires analyzing and modelling the temporal profiles of each individual variable (Teo et al., 2011).

This Research Topic aimed to investigate recent advancements in the field, focusing on two main areas: 1) evaluating how circadian rhythms and chronotype influence athletic performance or physical activity, and 2) examining how athletic performance or physical activity affects circadian rhythms and sleep patterns.

Kurtoğlu et al. conducted a cross-sectional study with 30 male participants aged 11–19 years, all diagnosed with mild Intellectual Disabilities (ID). Physical tests were administered in the morning (09:00–10:00 a.m.) and evening (05:00–06:00 p.m.). Also, chronotype was evaluated with a validated questionnaire. The results showed significant differences in performance based on the time of day, with higher performance levels observed in the evening compared to the morning. These findings highlight the importance of considering circadian rhythms when scheduling and planning physical activities to maximize benefits for individuals with ID. Moreover, a correlation between individual chronotype and performance parameters was found, thus highlighting the relevance of specific chronotype in individuals with ID.

Beníčková et al.’s review identified 925 articles examining the combined effect of circadian rhythm and menstrual cycle on women’s physical performance. After removing duplicates (n = 306), 619 studies were screened by title and abstract. 124 articles were excluded for not meeting authors’ criteria (e.g., reviews, conference papers, or books). Additionally, 84 studies involved animals, 28 were in a foreign language, and 374 were irrelevant. Two studies lacked full texts, and three had inadequate study designs.

Ultimately, Beníčková et al. included four studies in the review and employed various selection criteria. The most common criteria were having a regular menstrual cycle, not using hormonal contraception for at least 4, 6, or 12 months, being free from any sleep disorders, and not being pregnant or breastfeeding in the past 4 years. Only two of the studies highlighted the interaction between the time of day and menstrual cycle phase on physical performance. Specifically, isometric strength was greater in the afternoon during the mid-luteal phase, while maximum cycling power peaked in the afternoon during the mid-follicular phase. Due to the complexity of the Research Topic, heterogeneous physical activity protocols, and the different menstrual cycle phases to be taken into consideration, further studies are needed, and the matter is still in its infancy.

Ciorciari et al. studied 58 young soccer players aged 13–19 years to assess the influence of sleep and chronotype on aerobic performance. Physical tests were conducted in the morning (08:30 a.m.) and evening (06:00 p.m.), with participants categorized into three chronotypes and two sleep quality groups. The findings indicated that M-types performed better in the morning, while E-types excelled in the evening. Additionally, players with good sleep quality outperformed those with poor sleep quality in the evening aerobic endurance test. These results underscore the importance of considering the combination of chronotype and sleep quality when planning training schedules and competitions.

Finally, the study by Youngstedt theorized that shifting the early morning workout to later in the day could have beneficial effects in terms of reduction of sleep loss in endurance athletes, even though this hypothesis has not been tested. In fact, several potential barriers and drawbacks would need to be addressed, such as class schedules for collegiate athletes, tradition and coaches’ attitudes, logistics and safety reasons, and specificity of training time and performance. In this article, the author proposes several ways to test this hypothesis, which could be impactful in promoting restorative sleep, especially in a global context of poor sleep worldwide in modern society (Weaver et al., 2021).

## Final considerations

The importance of circadian rhythm on sport performance and physical activity is too essential to be ignored. Both the relevance of individual circadian features on physical performance, and the impact of physical activity on the circadian timing system remain inadequately characterized. This Research Topic has explored both aspects, expanding and critically reviewing available evidence. Future studies could be focused on biological variables linked to performance or physical activity such as perception of effort, heart rate variability, core body temperature patterns, hormones and menstrual cycle, recovery, cognitive performance relevant to physical activity, injuries, and fatigue. In addition, we believe it would be appropriate to study those living with intellectual disabilities and/or acute and chronic diseases in relation to sport performance, sleep, chronotype and circadian function (Montaruli et al., 2021; Castelli et al., 2023; Davies et al., 2023; Hughes et al., 2023).
